# Comparative Study on *In Vitro* Culture of Mouse Bone Marrow Mesenchymal Stem Cells

**DOI:** 10.1155/2018/6704583

**Published:** 2018-04-11

**Authors:** Yuxin Hu, Bin Lou, Xiafang Wu, Ruirui Wu, Huihui Wang, Lanyue Gao, Jingbo Pi, Yuanyuan Xu

**Affiliations:** ^1^Experimental Teaching Center, School of Public Health, China Medical University, Shenyang, Liaoning, China; ^2^Department of Hygiene Toxicology, School of Public Health, China Medical University, Shenyang, Liaoning, China

## Abstract

*In vitro* culture of mesenchymal stem cells (MSCs) from mouse bone marrow (BM) has been hampered because of the low yield of MSCs during isolation and the contamination of hematopoietic cells during expansion. The lack of specific mouse BM-MSC markers increases the difficulty. Several techniques have been reported to improve the purity and *in vitro* growth of mouse BM-MSCs. However, systematic report on comparison of characteristics in primary BM-MSCs between different culture conditions is rare. Here, we studied the effects of oxygen concentrations and initial medium replacement intervals, along with cell passages, on mouse BM-MSCs isolated with differential adhesion method. BM-MSCs exhibited elevated proliferative and clonogenic abilities in 5% oxygen compared with 10% and 21% oxygen, as well as a better expression of the MSC marker Sca-1. Adipogenic and osteogenetic differentiation of BM-MSCs can be observed in both 21% and 5% oxygen. Adipogenic differentiation appeared stronger under normoxia conditions. BM-MSCs showed increased proliferative capacity and adipogenic/osteogenetic differentiation potential when initial medium replacement interval was 4 days compared with 1 day. As passage number increased, cells were more MSC-like in morphology and in expression of surface markers (positive for CD29, CD44, and Sca-1 and negative for CD11b, CD19, and CD45). These data provide new insight into optimizing the culture method and understanding the biological characteristics of mouse BM-MSCs during *in vitro* expansion.

## 1. Introduction

Mesenchymal stem cells (MSCs) are undifferentiated cells with the ability to proliferate and the potential to differentiate into lineages of mesenchymal tissues, including the bone, cartilage, fat, tendon, muscle, and marrow stroma [1–5]. Bone marrow mesenchymal stem cells (BM-MSCs) can be isolated based on their feature of adherence to the plastic culture surface. Therefore, the method of differential adhesion is widely used to isolate BM-MSCs [2, 6–8]. To date, BM-MSCs have been successfully isolated and characterized from a number of species, including human [1, 5, 9], rat [10, 11], rabbit [12], goat [13], and dog [14]. An issue for this method is the contamination of hematopoietic stem cells (HSCs) in MSCs, because these two distinct types of somatic stem cells coexist in a unique niche in the bone marrow [15]. The isolation and purification of BM-MSCs from mouse are more difficult than those from human and other species, because mouse bone marrow-derived adherent cells are more heterogeneous and contain a high percentage of HSCs [16]. Several techniques have been applied to improve the purity of mouse BM-MSCs, including the use of low-density culture, frequent medium change, positive and negative selection, and combination of mechanical crushing and collagenase digestion [16–21]. Most of these methods have not gained widespread acceptance so far. Hence, a standardized, reliable, easy to perform, and similar to *in vivo* method is still in need to obtain high amounts of purified mouse BM-MSCs.

Surface markers have been used to isolate BM-MSCs or to assess the purity of BM-MSCs [2, 16, 22]. However, BM-MSC surface markers are highly species dependent. For example, BM-MSCs from C57BL/6 mouse express high levels of CD34 (primitive hematopoietic progenitor and endothelial cell marker) but no CD90 (thymocyte antigen). On the contrary, human MSCs were negative for CD34 but positive for CD90 [23, 24]. Even though BM-MSCs are from the same species, different strains showed varied profiles of surface markers. As reported previously, BM-MSCs from C57BL/6, DBA1, and FVB/N mice were positive for stem cell antigen-1 (Sca-1), but BALB/c mice were negative for Sca-1 [23]. A set of surface markers is related to proliferative capacity of MSCs. For example, Sca-1-positive MSCs showed enhanced proliferative capacity compared with Sca-1-negative MSCs [22]. Combination of surface markers has been applied to isolate and identify MSCs from the mouse bone marrow due to the lack of strain- and cell-specific markers. Generally, the identification of BM-MSCs is based on three characteristics including cell morphology, surface markers, and differentiation capability. The above three characteristics may be changed as the *in vitro* expansion prolongs [16, 22, 25]. In addition, changes in MSC surface markers during *in vitro* expansion were not consistent among individuals [26]. Therefore, possible alterations in expression of surface markers in freshly isolated and long-term cultured BM-MSCs require further investigations.

The value of physiological oxygen pressure in the bone marrow varies from 1% to 7% [27]. Although oxygen concentration has been recognized to exert an important impact on characteristics of BM-MSCs, including proliferation, plasticity, and differentiation [22, 27–29], BM-MSCs are mostly cultured in 21% oxygen condition [2, 8, 17], which leads to diminished growth potential and typical senescence of BM-MSCs after a few passages [28]. Mouse BM-MSCs serve as an ideal tool to explore the cell biology and the therapeutic potential of MSCs [16]. The lack of a standard and consistent method to isolate and culture mouse BM-MSCs restricts the study of basic aspects in MSC biology [17].

The aim of this study is to optimize the *in vitro* expansion conditions of BM-MSCs from the widely used C57BL/6 mouse. In the present study, we found that culture in hypoxia promoted cell proliferation and clonogenicity, while culture in normoxia appeared to favor adipogenic differentiation of BM-MSCs. Prolonged initial medium replacement interval (4 days compared with 1 day) improved proliferation, clonogenicity, and differentiation potential of BM-MSCs. In addition, under our isolation and expansion conditions, the differentiation potential of BM-MSCs was not hampered by long-term *in vitro* culture.

## 2. Materials and Methods

### 2.1. Isolation and Culture Conditions for Mouse BM-MSCs

To isolate mouse BM-MSCs, 6- to 8-week-old male C57BL/6 mice (Model Animal Research Center of Nanjing University, Nanjing, China) were sacrificed. Femurs and tibias from mice were dissected, and the bone marrow was flushed with Dulbecco's phosphate-buffered solution (DPBS; Biological Industries, Israel). Bone marrow-derived cells were filtered through 70 *μ*m cell strainer (BD Falcon, USA), collected by centrifugation at 300 ×g for 10 min, and suspended in 1 ml Dulbecco's modified Eagle's medium (DMEM; Biological Industries) containing 1500 mg/l D-glucose supplemented with 20% fetal bovine serum (FBS; Biological Industries), 1% penicillin/streptomycin (P/S; Biological Industries), and 1% glutamine (Thermo Scientific, Waltham, USA) (BM-MSC medium). Cells were seeded in the 25 cm^2^ flask at a density of 1.6 × 10^6^ cells/cm^2^ and incubated in a humidified incubator at 37°C with 21%, 10%, or 5% oxygen. After 1 day or 4 days, nonadherent cells were removed by washing with DPBS twice, and 5 ml fresh BM-MSC medium was added. The primary culture was approximately 80% confluent in one week. For subculture, cells were detached with 0.25% trypsin-ethylene diamine tetra-acetic acid (Gibco Invitrogen, USA) and seeded at 1 × 10^4^ cells/cm^2^ in BM-MSC medium. Then medium was refreshed every 4 days. Experiments were carried out under protocols approved by China Medical University (authorization reference number 14008M). All methods were performed in accordance with the relevant guidelines and regulations.

### 2.2. Characterization of Cell Surface Markers

Cells (passage (P)2, P8, and P13) at 80% to 90% confluence were trypsinized and collected by centrifugation. Then 1 × 10^5^ cells were incubated with fluorescence-labeled antibodies against CD29 (integrin beta-1), CD44 (receptor for hyaluronate and osteopontin), Sca-1, CD11b (macrophage and monocyte marker), CD19 (antigen receptor of B lymphocytes), and CD45 (pan-leukocyte marker) at 4°C for 10 min in the dark. The antibodies mentioned above were purchased from Miltenyi (Bergisch Gladbach, Germany). Expression of the above surface markers was determined with flow cytometer (Canto II, Becton, Dickinson and Company, USA).

### 2.3. Cell Proliferative Capacity and Colony-Forming Unit Assay

Cell proliferative capacity was assessed by Colorimetric Cell Counting Kit (CCK8, Vazyme Biotech, Nanjing, China). Cells cultured in 21%, 10%, or 5% oxygen conditions were trypsinized and seeded at a density of 1 × 10^4^ cells/well in the 96-well plate. The CCK8 assays were performed on day 1, 3, 5, 7, or 9. The absorbance at 450 nm was read using a Synergy H1 microplate reader (Biotek, Vermont, USA). The final value for absorption (A) was calculated by the A value of the test well minus A value of the reagent background.

For colony-forming unit (CFU) assay, 1 × 10^4^ cells/well were seeded in the 6-well plate and incubated in 21%, 10%, or 5% oxygen condition for 10 days. Culture medium was changed on days 3 and 7. On the 10th day, cells were fixed in 4% paraformaldehyde (PFA) for 15 min and stained with crystal violet (Solarbio, Beijing, China) for 30 min in the dark. After washing with DPBS twice, colonies with an area above 1 mm^2^ were counted.

### 2.4. Adipogenic and Osteogenic Differentiation

For *in vitro* adipogenesis, cells were grown in BM-MSC medium until a confluent layer was formed, alternatively cultured in adipogenic induction medium (DMEM was supplemented with 10% FBS, 1% P/S, 1% glutamine, 1 *μ*M dexamethasone, 0.125 mM indomethacin, 0.5 mM 3-isobutyl-1-methyl-xanthine, and 5 *μ*g/ml insulin) (Sigma, St. Louis, USA) for 3 days and then in adipogenic maintenance medium (DMEM was supplemented with 10% FBS, 1% P/S, 1% glutamine, and 1 *μ*M dexamethasone) for 1 day. After two weeks, cells were fixed with 4% PFA for 15 min and stained with Oil Red O (Sigma). Lipid accumulations were quantified by elution of Oil Red O in 60% isopropanol for 10 min at room temperature. The absorbance of the extracted dye was detected at 515 nm using a Synergy H1 microplate reader. The cells were also used for analysis of adipogenic gene expression by reverse transcript quantitative polymerase chain reaction (RT-qPCR).

For *in vitro* osteogenesis, cells were grown until 80% confluence and incubated in osteogenic induction medium (DMEM was supplemented with 10% FBS, 1% P/S, 1% glutamine, 0.1 *μ*M dexamethasone, 10 mM *β*-glycerophosphate disodium salt hydrate, and 50 *μ*M L-ascorbic acid 2-phosphate sesquimagnesium salt hydrate) (Sigma). The medium was changed every 2 days. After induction for 7 days, quantitative alkaline phosphatase (ALP) measurement was performed as described in the manufacturer's instruction (Beyotime, Shanghai, China). The absorbance was detected at 405 nm using a Synergy H1 microplate reader. After induction for 14 days, cells were fixed in 4% PFA for 15 min and stained with Alizarin Red S (Sigma) for 30 min at 37°C.

### 2.5. RT-qPCR

Total RNA was extracted with TRIzol Reagent (Cwbio, Beijing, China). The quantity and quality of the isolated RNA were evaluated by Thermo Scientific NanoDrop 2000 spectrophotometer (Waltham, USA). The RNA was reversely transcribed into cDNA with reverse transcriptase kit (Takara, Dalian, China) according to the manufacturer's instruction. cDNA amplification was determined with SYBR Green mix from Takara (Dalina, China) and QuantStudio™ 6 Real-Time PCR System from Applied Biosystems (USA). Primer sequences are shown in Table 1. Data were analyzed with the delta-delta cycle time method.

### 2.6. Statistical Analysis

All statistical analyses were performed using GraphPad Prism 5 (GraphPad Software, San Diego, CA). Statistical significance was defined as *p* < 0.05. Data are expressed as mean ± standard deviation (SD). For comparisons between two groups, Student's *t*-test was performed. Statistical differences between multiple groups were determined by analysis of variance (ANOVA), followed with Bonferroni post hoc comparison test.

## 3. Results

### 3.1. Cellular Morphology and Surface Markers of BM-MSCs Cultured in Normoxia and Hypoxia

**Figure 1:**
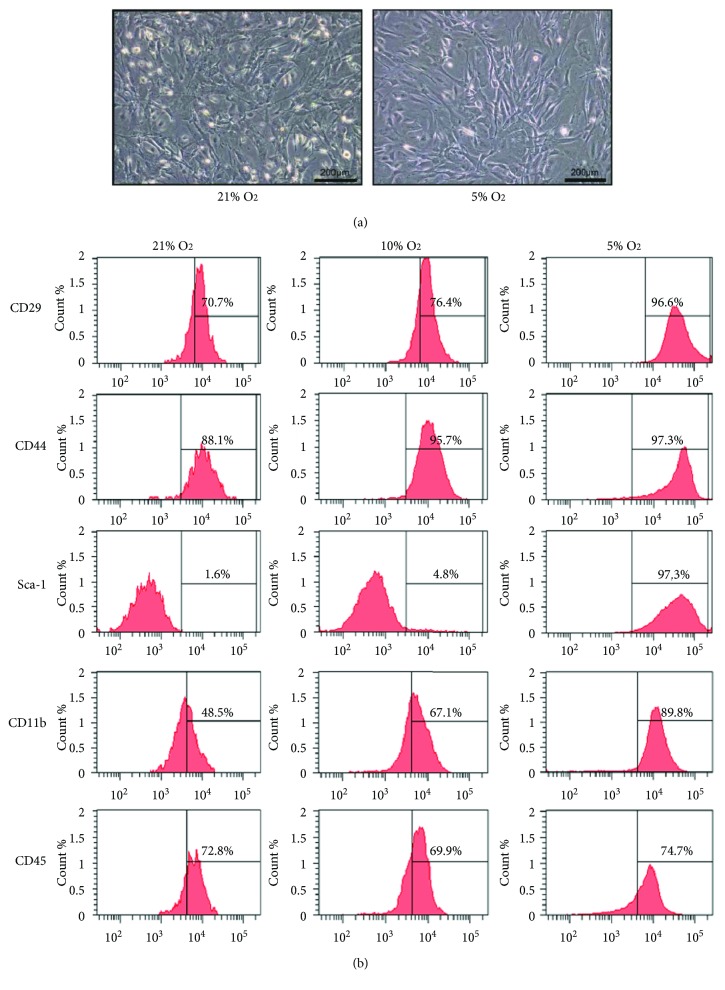
Effects of various oxygen concentrations on the cellular morphology and surface markers of BM-MSCs. (a) Representative image of BM-MSCs cultured in 21% and 5% oxygen conditions (×100, scale bar = 200 *μ*m). (b) The expression of CD29, CD44, Sca-1, CD11b, and CD45 surface markers determined by flow cytometry in BM-MSCs cultured in different oxygen concentrations (21%, 10%, and 5%).

BM-MSCs were isolated by their adhesion to cell culture surfaces and cultured in normoxia (21% oxygen) and hypoxia (10% and 5% oxygen). At P6, cells cultured in normoxia consisted of a heterogeneous cell population including convex round, convex spindled, and flattened spindled morphology. The cellular morphology of BM-MSCs in 5% oxygen was obviously more flattened spindle-shaped and less convex compared with BM-MSCs in 21% oxygen (Figure 1(a)). Flow cytometric analysis was performed on cells cultured in different oxygen conditions with markers commonly used to characterize mouse BM-MSCs, namely, CD29, CD44, Sca-1, CD11b, and CD45 [8, 29]. The proportions of CD29- and CD44-positive cells, especially Sca-1-positive cells, were increased as oxygen concentrations decreased, which were more than 95% in 5% oxygen but less than 5% in 21% or 10% oxygen. Unexpectedly, CD11b, which is supposed to be negative in BM-MSCs, was expressed in a big fraction of cultured cells. In addition, the number of CD11b-negative cells was increased in hypoxia (Figure 1(b)). This indicates the contamination of hematopoietic cells in MSCs at earlier passages. Increased cell passage number could result in a significant reduction of contaminating cells [22].

### 3.2. Proliferative Capacity and Differentiation Potential of BM-MSCs in Normoxia and Hypoxia

**Figure 2:**
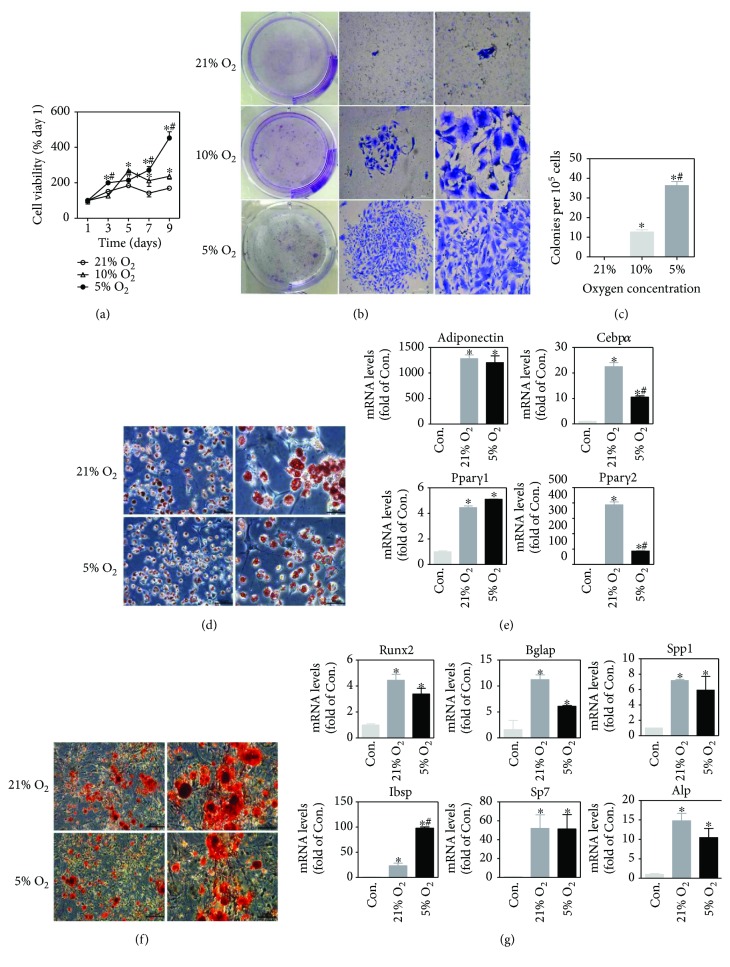
The effects of oxygen concentration on proliferation capacity and differentiation potential of BM-MSCs. (a) Comparison of cell viability in different oxygen concentrations as determined by CCK8. (b) Representative images for colonies in each group in CFU assay (from left to right, the view from one well of the six-well plate, ×40 with scale bar = 500 *μ*m, and ×100 with scale bar = 200 *μ*m). (c) Quantification of CFU assay. Colonies with an area above 1 mm^2^ were counted. (d) Representative images for Oil Red O staining in 21% and 5% oxygen conditions (from left to right, ×200 with scale bar = 100 *μ*m, ×400 with scale bar = 50 *μ*m). (e) The expression of adipogenic differentiation-related genes of BM-MSCs after differentiation induction in 21% and 5% oxygen conditions. (f) Representative images for Alizarin Red S staining in 21% and 5% oxygen conditions (from left to right, ×100 with scale bar = 200 *μ*m, ×200 with scale bar = 100 *μ*m). (g) The expression of osteogenetic differentiation-related genes of BM-MSCs after differentiation induction in 21% and 5% oxygen conditions. P15 and P11 BM-MSCs were used for adipogenic and osteogenic differentiation, respectively. Results are expressed as mean ± SD from three independent measurements. ^∗^*p* < 0.05 versus Con. group; ^#^*p* < 0.05 versus 21% oxygen.

The effect of oxygen tension on proliferation and clonogenicity of BM-MSCs was examined with CCK8 and CFU methods, respectively. As shown in Figure 2(a), proliferative capacity of BM-MSCs cultured in 10% oxygen was significantly higher than that cultured in normoxia (21% oxygen) from day 5 to day 9 after being seeded in the 96-well plate. On the 3rd, 7th, and 9th day, BM-MSCs cultured in 5% oxygen condition generated a remarkably higher proliferative capacity in comparison to those cultured in 10% or 21% oxygen condition. Consistently, colonies in 5% oxygen were larger in size and denser in cell number than the other two oxygen concentrations (Figure 2(b)). In addition, BM-MSCs cultured in 5% oxygen condition generated a significantly higher number of CFUs in comparison to 21% or 10% oxygen condition (Figure 2(c)). All data suggest an oxygen concentration-dependent decline in cell proliferation and clonogenicity.

Adipogenic and osteogenic differentiation potential of BM-MSCs cultured in different oxygen conditions was tested at P15 and P11, respectively. As shown in Figures 2(d) and 2(f), lipid granules stained with Oil Red O and calcium nodule depositions stained with Alizarin Red S were observed in BM-MSCs in both 21% and 5% oxygen condition after differentiation induction. The expression of adipogenic differentiation-related genes (*Adiponectin*, *Cebpα*, *Pparγ1*, and *Pparγ2*) and osteogenetic differentiation-related genes (*Runx2*, *Bglap*, *Spp1*, *Ibsp*, *Sp7*, and *Alp*) was significantly increased when cells were cultured in differentiation medium in 21% and 5% oxygen condition (Figures 2(e) and 2(g)). BM-MSCs, which were initially expanded in 5% oxygen and subsequently induced to differentiate in 21% oxygen condition, appeared to gain enhanced adipogenic differentiation capacity compared with those induced to differentiate in 5% oxygen condition according to the expression of *Cebpα* and *Pparγ2* (Figure 2(e)). There was no big difference in osteogenic differentiation potential between 21% oxygen and 5% oxygen conditions according to the Alizarin Red S staining and the expression of osteogenetic differentiation-related genes (Figures 2(f) and 2(g)).

### 3.3. Effect of Initial Medium Replacement Interval on BM-MSC Characteristics

**Figure 3:**
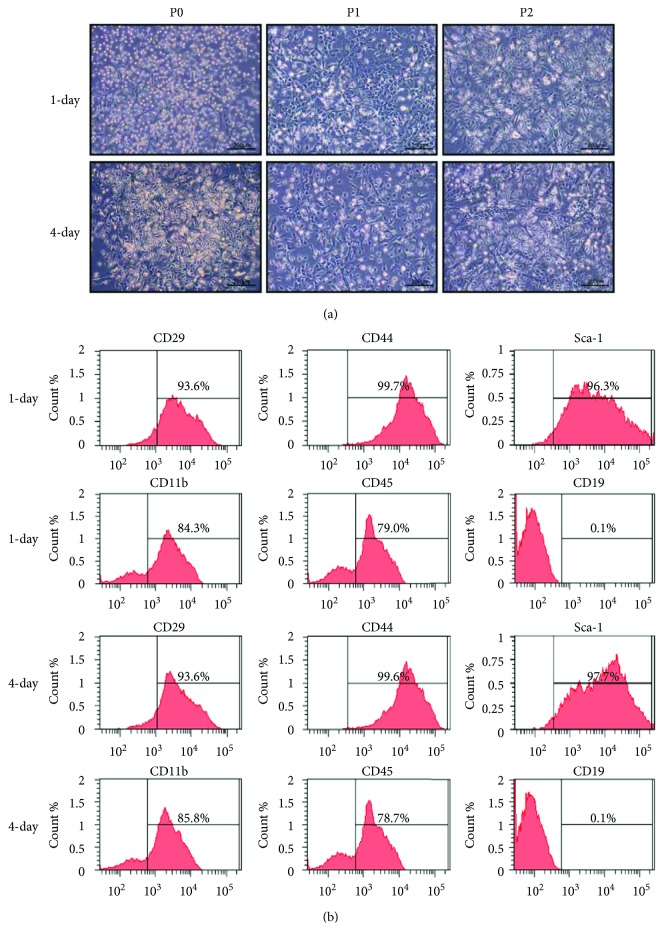
The effect of the different initial medium replacement intervals on cellular morphology and surface markers of BM-MSCs cultured in 5% oxygen condition. (a) Phase contrast images (×100 with scale bar = 200 *μ*m) of BM-MSCs at P0, P1, and P2. (b) The expression of CD29, CD44, Sca-1, CD11b, CD45, and CD19 surface markers of BM-MSCs at P2 determined by flow cytometry.

BM-MSCs were frequently isolated and purified by differential adhesion; however, initial medium replacement interval to remove the nonadherent cells was different [6, 7, 16, 22]. As shown in Figures 3(a) and 3(b), there is no big difference between 1-day and 4-day interval groups in cellular morphology or surface markers of BM-MSCs. Cells with spindle-like shape, typical for BM-MSCs, were predominant in the cultures from P0 to P2 under both conditions.

**Figure 4:**
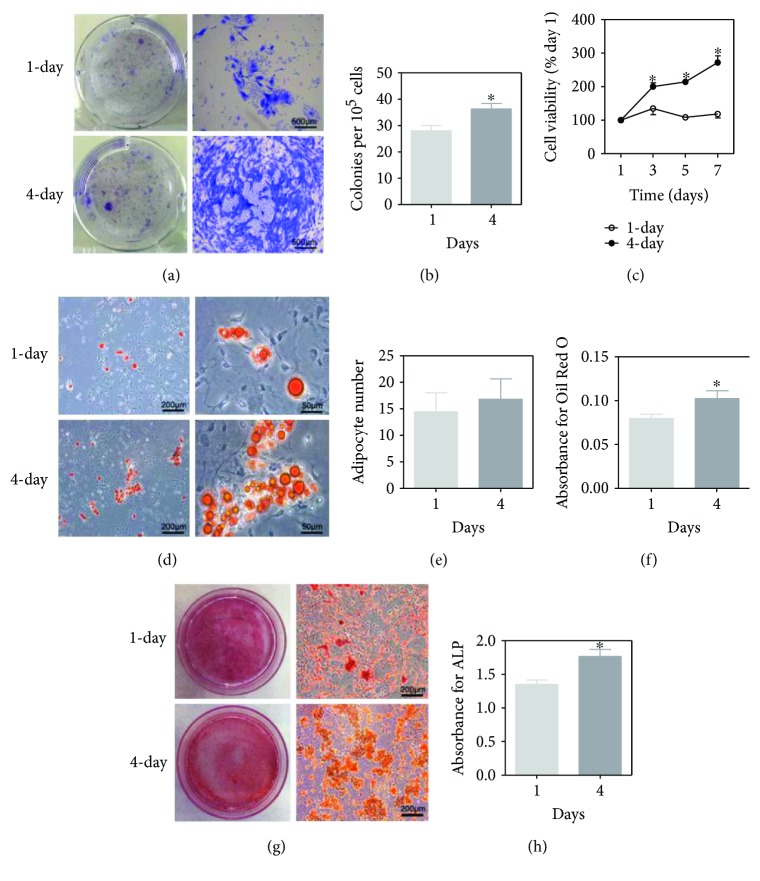
The effect of different initial medium replacement intervals on proliferation capacity and differentiation potential of BM-MSCs cultured in 5% oxygen condition. (a) Representative image of CFU assay (from left to right, the view from one well of the six-well plate, ×40 with scale bar = 500 *μ*m). (b) Quantification of clonogenicity of BM-MSCs by CFU assay. Colonies with an area above 1 mm^2^ were counted. (c) Cell viability determined by CCK8 assay. (d) Oil Red O staining after 2 weeks of adipogenic induction (from left to right, ×100 with scale bar = 200 *μ*m and ×400 with scale bar = 50 *μ*m). (e) The number of adipocytes within a microscopic field (×100) in 1-day or 4-day medium replacement interval group. (f) Quantification of Oil Red O staining. (g) Images of Alizarin Red S staining (from left to right, the view from one 35 mm dish, ×100 with scale bar = 200 *μ*m). (h) Quantification of ALP expression. Results are expressed as mean ± SD from three independent measurements. ^∗^*p* < 0.05 versus 1-day group.

Next, we analyzed the cell proliferation and clonogenicity of BM-MSCs with different initial medium replacement intervals. Cells cultured with prolonged interval formed more and bigger (Figures 4(a) and 4(b)) colonies and showed a higher proliferative capacity, which reached the peak on the 7th day after seeding (Figure 4(c)).

Adipogenic and osteogenic differentiation potential of BM-MSCs was tested at P2 and P4, respectively. BM-MSCs gave rise to more lipid droplets in the 4-day interval group than the 1-day interval group as determined by Oil Red O staining after 2 weeks of adipogenic induction (Figures 4(d) and 4(e)). Consistently, the quantification results indicated that cells with 4-day initial medium replacement interval had a higher adipogenic differentiation potential (Figure 4(f)). There was no difference in osteogenic differentiation potential between different medium replacement intervals as indicated by Alizarin Red S staining when examined by naked eyes (left part of Figure 4(g)). However, it is clear under microscope that the number of calcifying nodules formed by BM-MSCs from the 4-day interval group was more than the 1-day interval group (right part of Figure 4(g)). In addition, ALP activity was higher in BM-MSCs from 4-day interval group compared with 1-day interval group on day 7 after osteogenic induction (Figure 4(h)).

### 3.4. Effect of Cell Passages on BM-MSC Characteristics

**Figure 5:**
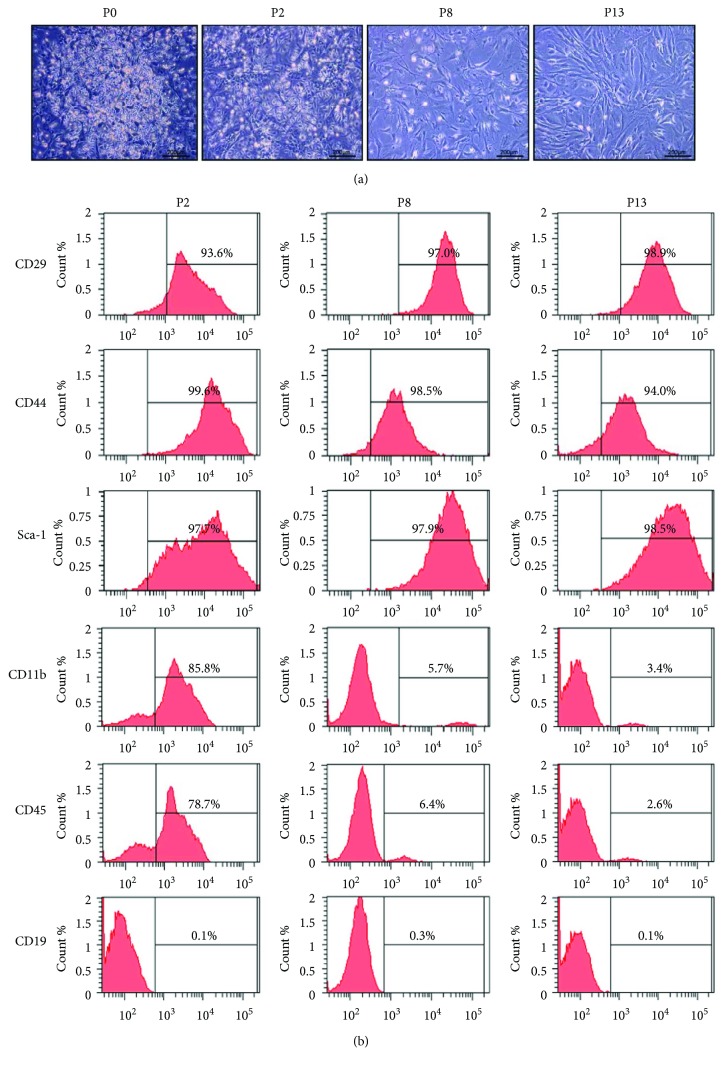
Morphology and cell surface markers of BM-MSCs at different passages cultured in 5% oxygen condition. (a) Phase contrast images of BM-MSCs at P0, P2, P8, or P13 (×100 with scale bar = 200 *μ*m). (b) The expression of CD29, CD44, Sca-1, CD11b, CD45, and CD19 surface markers of BM-MSCs at P2, P8, or P13 determined by flow cytometry.

Under the culture conditions optimized above (5% oxygen condition and 4-day initial medium replacement interval), cells usually remained highly morphologically heterogeneous before P2, presenting with convex round, convex spindled, and flattened spindled shapes. The proportion of flattened spindle-shaped cells was gradually elevated with the increase of cell passage number. The morphological heterogeneity was not obvious until P6 (Figure 1(a)). The flattened cell type was predominant in cultures at P8 (Figure 5(a)).

We further investigated whether cell passages had an effect on the cell surface markers of BM-MSCs. BM-MSCs were positive for CD29, CD44, and Sca-1, but negative for CD19, no matter which passage they were at. However, BM-MSCs were positive for the macrophage marker CD11b and hematopoietic stem cell marker CD45 at the earlier passage (P2), but negative for CD11b and CD45 at the later passages (P8 and P13) (Figure 5(b)).

**Figure 6:**
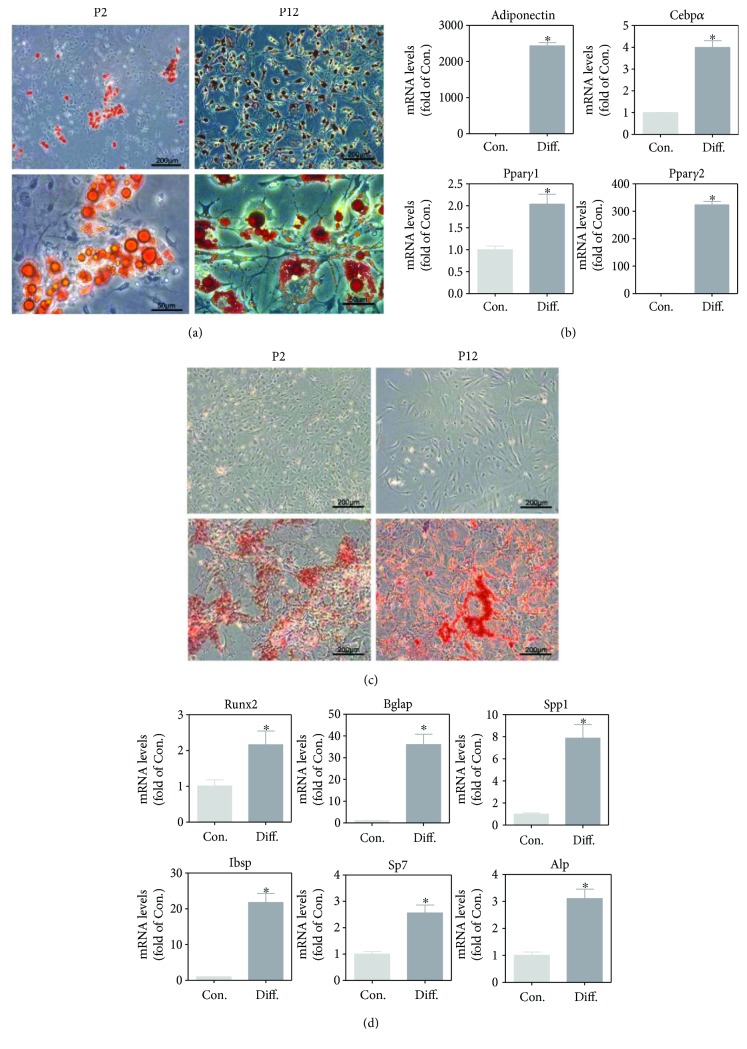
Adipogenic differentiation and osteogenetic differentiation at the earlier passage and the later passage of BM-MSCs cultured in 5% oxygen condition. (a) Representative images for Oil Red O staining at P2 and P12 (from upper to lower, ×100 with scale bar = 200 *μ*m, ×400 with scale bar = 50 *μ*m). (b) The expression of adipogenic differentiation-related genes of BM-MSCs at P12 in BM-MSCs cultured in BM-MSC medium (Con.) and adipogenic induction medium (Diff.). (c) Representative images for Alizarin Red S staining at P2 and P12 (×100 with scale bar = 200 *μ*m, from upper to lower: cultured in BM-MSC medium, and cultured in osteogenetic induction medium). (d) The expression of osteogenetic differentiation-related genes at P12 in BM-MSCs cultured in BM-MSC medium (Con.) and osteogenetic induction medium (Diff.). Results are expressed as mean ± SD from three independent measurements. ^∗^*p* < 0.05 versus Con. group.

Adipogenic and osteogenic differentiation potential was maintained in cells at earlier passage (P2) and later passage (P12). Lipid granules stained with Oil Red O (Figure 6(a)) and calcium nodule depositions stained with Alizarin Red S (Figure 6(c)) were observed in BM-MSCs at both P2 and P12. Consistently, the expression of adipogenic differentiation-related genes (*Adiponectin*, *Cebpα*, *Pparγ1*, and *Pparγ2*) and osteogenetic differentiation-related genes (*Runx2*, *Bglap*, *Spp1*, *Ibsp*, *Sp7*, and *Alp*) was significantly increased when cells were cultured in adipogenic or osteogenic medium for two weeks at P12 (Figures 6(b) and 6(d)). Thus, under our culture conditions, the adipogenic and osteogenic differentiation potential of BM-MSCs was not hampered by the *in vitro* passage.

## 4. Discussion

BM-MSCs have generated a great deal of research interest owing to their intrinsic ability to self-renew and differentiate into various types of functional cells [16]. Mouse BM-MSCs provide valuable information on cell biology and potential function of MSCs. However, isolation and purification of BM-MSCs from mouse are more difficult than from other species because of the low MSC frequencies and the high proportion of HSCs in the BM. Several techniques have been reported to improve the purity of mouse BM-MSCs (Table 2). However, the optimum operation about oxygen concentrations and initial medium replacement intervals, along with the cell passages, has not been standardized.

A key factor often ignored by researchers is that the physiological oxygen concentration in the bone marrow varies from 1% to 7%. Although low-oxygen culture conditions have been recognized to exert an important impact on BM-MSCs, mouse BM-MSCs are cultured in 21% oxygen condition in many studies [2, 7, 8, 17, 30–32]. Here, we compared cell proliferation, clonogenicity, and surface markers between 21%, 10%, and 5% oxygen conditions. Though we did not detect the oxygen concentration in the medium, which may be more relevant to the physiology of the cells, 5% oxygen condition is considered to mimic *in vivo* microenvironment in the bone marrow. When cultured in 5% oxygen condition, MSCs showed higher proliferation and clonogenicity than cultured in 21% and 10% oxygen conditions (Figure 2). Comparison of CFU-F frequencies between compact bone-derived cells cultured in 20% oxygen versus 5% oxygen reveals a sevenfold increase in the number of colonies when grown in low-oxygen conditions [33]. The proportion of Sca-1-positive cells was increased as the oxygen concentrations decreased (Figure 1(b)). The above results were in agreement with previous report [22], in which MSCs cultured in 2% oxygen required significantly reduced *ex vivo* expansion time, with significantly increased numbers of Sca-1-positive as well as Sca-1/CD44 double-positive cells [34]. Sca-1-positive cells had higher CFU-F frequencies and showed enhanced proliferation compared with Sca-1-negative cells [22]. Sca-1-positive-derived colony cells of trophoblast population could proliferate over many passages and were established as a stable cell line [35]. The proportion of Sca-1 cells homed to the heart was significantly greater in young than old chimeras, suggesting that Sca-1-positive cells contribute to proliferation and tissue regeneration [36]. Unexpectedly, the proportion of cells expressing CD11b which is supposed to be negative for BM-MSCs was increased as the oxygen concentration decreased at P1 (Figure 1(b)). CD11b is expressed on the surface of many leukocytes including monocytes, neutrophils, and natural killer cells, which exist in the bone marrow and blood [37]. Hypoxia culture condition may also benefit for CD11b-positive cells in short term. However, CD11b population shrank as cell passage number increased under our culture conditions. Normoxic conditions rapidly induced p53 expression and oxidative stress related to generation of mitochondrial reactive oxygen species (ROS), resulting in inhibition of cell proliferation of primary mouse BM-MSCs [38]. On the other hand, the expression of hypoxia-inducible factor (HIF) family members was dramatically increased in hypoxia [39]. HIF 1 alpha (HIF1*α*) improved viability and suppressed apoptosis of MSCs by repressing the expression of p53, phosphate-p53, and p21 in MSCs in 3% oxygen [40]. However, the proliferation of MSCs was inhibited through HIF1*α*-dependent regulation of p27 when cells were cultured at 1% oxygen [41]. Recently, low-oxygen environment has been applied to human MSC culture in more and more studies and found to improve MSC functions [27, 42–45]. Similar culture condition should be used for mouse BM-MSCs in order to better mimic the *in vivo* environment, which plays a major role in the regulation of cell homeostasis and function.

As common progenitor cells of adipocytes and osteoblasts, MSCs are delicately balanced for their differentiation commitment [46, 47]. Furthermore, the adipogenic and osteogenic differentiation of BM-MSCs in normoxia and hypoxia were evaluated. Both conditions are suitable for MSC differentiation. Normoxia appeared to provide a more favorable environment for adipogenic differentiation according to the expression of differentiation-related genes. These data support the previous finding that hypoxic condition reversibly decreased adipogenic differentiation [45, 48]. The adipogenic master transcription factor PPAR*γ*2 was repressed in hypoxia, which is HIF1*α* dependent [49]. In contrast, another report showed that hypoxia-mediated decrease of adipogenic differentiation was HIF independent [45]. Hypoxia provides a milieu that extends cellular lifespan and furthermore is beneficial for the stemness and self-renewal of MSCs [50], whereas normoxia is benefit for the adipogenic differentiation of BM-MSCs. According to the Alizarin Red S staining and the expression of osteogenetic differentiation-related genes (Figures 2(f) and 2(g)), there was no big difference in osteogenic differentiation potential in normoxia and hypoxia. MSCs have been demonstrated to play a role in tissue repair and regeneration. During tissue repair, MSCs can migrate to the injured tissues, secreting the immunomodulatory factors and differentiating into the special cells. The capacity to differentiate into multiple cell lineages has seen MSCs actively explored for tissue repair [51]. The oxygen pressure of differentiation niche may be different from the bone marrow depending on the specific situation. Therefore, microenvironment characteristics should be investigated when studying the function of BM-MSCs.

Several techniques, such as magnetic-activated cell sorting and flow cytometry sorting, have been described to improve the purity of mouse BM-MSCs from the method of differential adhesion [16–18, 22]. Most of these methods have not gained widespread acceptance so far because of the high expense, technical difficulties, and great damage to cells. The differential adhesion is a common method; however, the initial medium replacement intervals to remove the nonadherent cells are different, which vary from 3 h to 96 h [6, 7, 16, 22, 32]. We chose 1-day and 4-day intervals to remove the nonadherent cells. There was no difference in cellular morphology or MSC surface markers (Figure 3). Of interest, the latter was shown to increase cell proliferation and clonogenicity, as well as the ability of adipogenic and osteogenic differentiation than the former (Figure 4). In Figure 4(a), plates in the 4-day group contained distinct colonies of fibroblastic cells that vary in size and composition, with small numbers of hematopoietic cells interspersed between the colonies. Cultures with these characteristics typically produce good yields of MSCs (>15%) [52]. Earlier initial medium replacement helps prevent adherence of many non-MSCs, such as the hematopoietic populations, to the culture dish. However, it results in the loss of a big fraction of BM-MSCs as well. Once the initial density of BM-MSCs is too low, cell proliferation and clonogenicity can be influenced. In addition, later initial medium replacement may provide an *in vitro* culture condition close to *in vivo* microenvironment with complex cell mix, especially in the first few passages, and thus benefit for the expansion and function of BM-MSCs. The optimum initial medium replacement interval for isolation of mouse BM-MSCs with differential adhesion method and the underlying mechanisms should be further investigated.

CD29, CD44, CD81, CD90, CD106, and Sca-1, among others, are routinely used for the definition of mouse BM-MSCs [16, 17, 22]. Unfortunately, these markers are not BM-MSC specific. Thus, the negative surface markers, such as CD11b, CD31, CD45, CD48, CD90, CD106, CD117, and CD135, should be described together [17, 19]. It is noteworthy that some surface markers of BM-MSCs are different between mouse and human [23]. Furthermore, surface markers of mouse BM-MSCs were not consistently used in most studies. It has been debated that BM-MSC surface markers may vary among strains of inbred mice [19, 23]. Even in the same strain, for example, BALB/c, the reports are controversial [19, 23]. In the present study, we used flow cytometry to trace the profile of surface markers on cultured BM-MSCs at different cell passages. The surface markers are changing with the increase of cell passage, which may be a reason for the conflicting reports on the expression of BM-MSC surface markers. BM-MSCs from C57BL/6 mice were positive for CD29, CD44, and Sca-1, but negative for CD11b, CD19, and CD45 at P8 and P13 after a few passages (Figure 5(b)), which is consistent with the widely accepted conception. The profile of these markers suggested that a relatively high purified BM-MSC population was achieved at P8. The positive or negative selection technique has been used to improve the purity of mouse BM-MSCs from plastic adhesion [16, 17, 25, 53]. However, there are several defects of the technique. First, cultured BM-MSCs show a different surface marker profile when compared to the freshly isolated primary cells [54]. Secondly, the exact composition of surface markers may not be known explicitly because there are no specific markers. Hence, it is difficult to make clear whether different characteristics of *in vitro* cultured BM-MSCs is due to different selection markers. Thirdly, positive or negative selection is high cost, time-consuming, and cell damaging. It seems that isolation by differential adhesion with properly prolonged cell-adherent interval and culture in hypoxia provides an easy-to-perform and low-cost method to achieve mouse BM-MSCs. Furthermore, the obtained BM-MSCs exhibited typical MSC morphology and differentiation ability, though not typical surface markers at the earlier cell passage. These data suggest that our current knowledge on putative surface markers is incomplete. A better understanding of MSC markers and their functions is needed to properly use surface marker profile as a predictive tool for cell purity and behavior both *in vitro* and in the clinic [55].

The present study has successfully generated and characterized the mouse BM-MSCs from C57BL/6 with optimized isolation and expansion conditions based on the oxygen concentration and the initial medium replacement interval. We demonstrated that hypoxic condition promoted self-renewal of BM-MSCs. In addition, BM-MSCs cultured under our conditions could subsequently be successfully stimulated to differentiate in 21% and 5% oxygen conditions and meanwhile present with a more MSC-like morphology and profile of surface markers as cell passage increased.

## Figures and Tables

**Table 1:** Primer sequences for reverse transcript quantitative PCR (RT-qPCR).

Gene	Sense	Antisense
*Gapdh*	GTATGACTCCACTCACGGCAAA	GGTCTCGCTCCTGGAAGATG
*Adiponectin*	GCTCAGGATGCTACTGTTGCAA	AACGTCATCTTCGGCATGACT
*Cebpα*	CGCAAGAGCCGAGATAAAGC	CGGTCATTGTCACTGGTCAACT
*Pparγ1*	GGTGAACCACTGATATTCAGGACA	TGTGTCAACCATGGTAATTTCAGT
*Pparγ2*	TGAGCACTTCACAAGAAATTACC	TGCGAGTGGTCTTCCATCAC
*Runx2*	GACTGTGGTTACCGTCATGGC	ACTTGGTTTTTCATAACAGCGGA
*Bglap*	GGCCCTGAGTCTGACAAAGC	GCTCGTCACAAGCAGGGTTAA
*Spp1*	CCCATCTCAGAAGCAGAATCTCC	TTCATCCGAGTCCACAGAATCC
*Ibsp*	CCACACCCCAAGCACAGACT	CTTTCTGCATCTCCAGCCTTCT
*Sp7*	GGTCCAGGCAACACACCTAC	GGTAGGGAGCTGGGTTAAGG
*Alp*	GCTCTCCGAGATGGTGGA	AGCCTGCTTGGCCTTACC

**Table 2:** Summary of studies describing isolate methods and *in vitro* culture conditions for mouse BM-MSCs.

Isolate method	Operation	Oxygen concentration	Initial medium replacement interval	Reference
Differential adhesion of the bone marrow	BM was flushed out from the bone cavity of femurs and tibias	Not mentioned	72 h	[25, 56]
	BM was flushed out from the bone cavity of femurs and tibias	Not mentioned	48 h	[8, 31, 57]
	BM was flushed out from the bone cavity of femurs and tibias	Not mentioned	24 h	[7, 30]
	BM was flushed out from the bone cavity of femurs and tibias	Not mentioned	3 h	[32]
	Frequent medium change and treatment of the primary cultures with trypsin were used to purify the BM-MSCs	Not mentioned	72 h	[19]
	Adapted centrifuge tubes were used to collect the bone marrow	Not mentioned	24 h	[23]
	Frequent medium change and the diminishing trypsinization time were used to purify the BM-MSCs	Not mentioned	8 h	[21]
Density gradient centrifugation	BM was flushed out from the tibia and femur into lymphocyte separation medium	Not mentioned	72 h	[58]
	BM was loaded on 2 ml lymphodex and centrifuged at 350 ×g for 15 min	Not mentioned	168 h	[20]
Immunomagnetic bead cell sorting	Anti-CD11b-, CD34-, and CD45-conjugated dynabeads were used for immunodepletion	Not mentioned	24 h or 48 h	[16]
	The magnetic beads conjugated to anti-CD11b, anti-CD34, and anti-CD45 antibodies were used for immunodepletion	Not mentioned	48 h	[17]
Fluorescence-activated cell sorting	Cells isolated from BM were labeled with monoclonal antibodies and sorted with FACSAria II	21%, 5%, or 2%	72 h or 96 h	[22]

## References

[1] Pittenger M. F., Mackay A. M., Beck S. C. (1999). Multilineage potential of adult human mesenchymal stem cells. *Science*.

[2] Qian K., Xu H., Dai T., Shi K. (2015). Effects of Tanshinone IIA on osteogenic differentiation of mouse bone marrow mesenchymal stem cells. *Naunyn-Schmiedeberg's Archives of Pharmacology*.

[3] Zhou N., Hu N., Liao J. Y. (2015). HIF-1*α* as a regulator of BMP2-induced chondrogenic differentiation, osteogenic differentiation, and endochondral ossification in stem cells. *Cellular Physiology and Biochemistry*.

[4] Deng P., Chen Y., Ji N. (2015). Cysteine dioxygenase type 1 promotes adipogenesis via interaction with peroxisome proliferator-activated receptor gamma. *Biochemical and Biophysical Research Communications*.

[5] Ghoniem A. A., Açil Y., Wiltfang J., Gierloff M. (2015). Improved adipogenic *in vitro* differentiation: comparison of different adipogenic cell culture media on human fat and bone stroma cells for fat tissue engineering. *Anatomy & Cell Biology*.

[6] Alcayaga-Miranda F., Cuenca J., Luz-Crawford P. (2015). Characterization of menstrual stem cells: angiogenic effect, migration and hematopoietic stem cell support in comparison with bone marrow mesenchymal stem cells. *Stem Cell Research & Therapy*.

[7] He X., Wang H., Jin T., Xu Y., Mei L., Yang J. (2016). TLR4 activation promotes bone marrow MSC proliferation and osteogenic differentiation via Wnt3a and Wnt5a signaling. *PLoS One*.

[8] Li P., Yang Y. M., Sanchez S. (2016). Deubiquitinase MYSM1 is essential for normal bone formation and mesenchymal stem cell differentiation. *Scientific Reports*.

[9] Zaim M., Karaman S., Cetin G., Isik S. (2012). Donor age and long-term culture affect differentiation and proliferation of human bone marrow mesenchymal stem cells. *Annals of Hematology*.

[10] Chen L. B., Jiang X. B., Yang L. (2004). Differentiation of rat marrow mesenchymal stem cells into pancreatic islet beta-cells. *World Journal of Gastroenterology*.

[11] Ridzuan N., Al Abbar A., Yip W. K., Maqbool M., Ramasamy R. (2016). Characterization and expression of senescence marker in prolonged passages of rat bone marrow-derived mesenchymal stem cells. *Stem Cells International*.

[12] Wang Y., Huang X., Tang Y., Lin H., Zhou N. (2016). Effects of panax notoginseng saponins on the osteogenic differentiation of rabbit bone mesenchymal stem cells through TGF-*β*1 signaling pathway. *BMC Complementary and Alternative Medicine*.

[13] Li P. Z., Yan G. Y., Han L. (2017). Overexpression of *STRA8*, *BOULE*, and *DAZL* genes promotes goat bone marrow–derived mesenchymal stem cells in vitro transdifferentiation toward putative male germ cells. *Reproductive Sciences*.

[14] Muir P., Hans E. C., Racette M. (2016). Autologous bone marrow-derived mesenchymal stem cells modulate molecular markers of inflammation in dogs with cruciate ligament rupture. *PLoS One*.

[15] Méndez-Ferrer S., Michurina T. V., Ferraro F. (2010). Mesenchymal and haematopoietic stem cells form a unique bone marrow niche. *Nature*.

[16] Xu S., De Becker A., Van Camp B., Vanderkerken K., Van Riet I. (2010). An improved harvest and *in vitro* expansion protocol for murine bone marrow-derived mesenchymal stem cells. *Journal of Biomedicine and Biotechnology*.

[17] Baddoo M., Hill K., Wilkinson R. (2003). Characterization of mesenchymal stem cells isolated from murine bone marrow by negative selection. *Journal of Cellular Biochemistry*.

[18] Abdallah B. M., Al-Shammary A., Skagen P. (2015). CD34 defines an osteoprogenitor cell population in mouse bone marrow stromal cells. *Stem Cell Research*.

[19] Nadri S., Soleimani M., Hosseni R. H., Massumi M., Atashi A., Izadpanah R. (2007). An efficient method for isolation of murine bone marrow mesenchymal stem cells. *The International Journal of Developmental Biology*.

[20] Eslaminejad M. B., Nikmahzar A., Taghiyar L., Nadri S., Massumi M. (2006). Murine mesenchymal stem cells isolated by low density primary culture system. *Development, Growth & Differentiation*.

[21] Soleimani M., Nadri S. (2009). A protocol for isolation and culture of mesenchymal stem cells from mouse bone marrow. *Nature Protocols*.

[22] Baustian C., Hanley S., Ceredig R. (2015). Isolation, selection and culture methods to enhance clonogenicity of mouse bone marrow derived mesenchymal stromal cell precursors. *Stem Cell Research & Therapy*.

[23] Peister A., Mellad J. A., Larson B. L., Hall B. M., Gibson L. F., Prockop D. J. (2004). Adult stem cells from bone marrow (MSCs) isolated from different strains of inbred mice vary in surface epitopes, rates of proliferation, and differentiation potential. *Blood*.

[24] Chen Y. H., Chung C. C., Liu Y. C. (2016). Enhancer of zeste homolog 2 and histone deacetylase 9c regulate age-dependent mesenchymal stem cell differentiation into osteoblasts and adipocytes. *Stem Cells*.

[25] da Silva Meirelles L., Nardi N. B. (2003). Murine marrow-derived mesenchymal stem cell: isolation, *in vitro* expansion, and characterization. *British Journal of Haematology*.

[26] Kundrotas G., Gasperskaja E., Slapsyte G. (2016). Identity, proliferation capacity, genomic stability and novel senescence markers of mesenchymal stem cells isolated from low volume of human bone marrow. *Oncotarget*.

[27] Hung S. C., Pochampally R. R., Hsu S. C. (2007). Short-term exposure of multipotent stromal cells to low oxygen increases their expression of CX3CR1 and CXCR4 and their engraftment in vivo. *PLoS One*.

[28] Jin Y., Kato T., Furu M. (2010). Mesenchymal stem cells cultured under hypoxia escape from senescence via down-regulation of p16 and extracellular signal regulated kinase. *Biochemical and Biophysical Research Communications*.

[29] Shi S., Wu X., Wang X. (2016). Differentiation of bone marrow mesenchymal stem cells to cardiomyocyte-like cells is regulated by the combined low dose treatment of transforming growth factor-*β*1 and 5-azacytidine. *Stem Cells International*.

[30] Borg D. J., Weigelt M., Wilhelm C. (2014). Mesenchymal stromal cells improve transplanted islet survival and islet function in a syngeneic mouse model. *Diabetologia*.

[31] Cho K. A., Woo S. Y., Seoh J. Y., Han H. S., Ryu K. H. (2012). Mesenchymal stem cells restore CCL_4_-induced liver injury by an antioxidative process. *Cell Biology International*.

[32] Nam Y. S., Kim N., Im K. I., Lim J. Y., Lee E. S., Cho S. G. (2015). Negative impact of bone-marrow-derived mesenchymal stem cells on dextran sulfate sodium-induced colitis. *World Journal of Gastroenterology*.

[33] Short B., Wagey R. (2013). Isolation and culture of mesenchymal stem cells from mouse compact bone. *Methods in Molecular Biology*.

[34] Valorani M. G., Germani A., Otto W. R. (2010). Hypoxia increases Sca-1/CD44 co-expression in murine mesenchymal stem cells and enhances their adipogenic differentiation potential. *Cell and Tissue Research*.

[35] Natale B. V., Schweitzer C., Hughes M. (2017). Sca-1 identifies a trophoblast population with multipotent potential in the mid-gestation mouse placenta. *Scientific Reports*.

[36] Li S. H., Sun L., Yang L. (2017). Young bone-marrow Sca-1^+^ stem cells rejuvenate the aged heart and improve function after injury through PDGFR*β*-Akt pathway. *Scientific Reports*.

[37] Kawai K., Tsuno N. H., Matsuhashi M. (2005). CD11b-mediated migratory property of peripheral blood B cells. *The Journal of Allergy and Clinical Immunology*.

[38] Boregowda S. V., Krishnappa V., Chambers J. W. (2012). Atmospheric oxygen inhibits growth and differentiation of marrow-derived mouse mesenchymal stem cells via a p53-dependent mechanism: implications for long-term culture expansion. *Stem Cells*.

[39] Prado-Lòpez S., Duffy M. M., Baustian C. (2014). The influence of hypoxia on the differentiation capacities and immunosuppressive properties of clonal mouse mesenchymal stromal cell lines. *Immunology & Cell Biology*.

[40] Lv B., Li F., Fang J. (2017). Hypoxia inducible factor 1*α* promotes survival of mesenchymal stem cells under hypoxia. *American Journal of Translational Research*.

[41] Kumar S., Vaidya M. (2016). Hypoxia inhibits mesenchymal stem cell proliferation through HIF1*α*-dependent regulation of P27. *Molecular and Cellular Biochemistry*.

[42] Paquet J., Deschepper M., Moya A., Logeart-Avramoglou D., Boisson-Vidal C., Petite H. (2015). Oxygen tension regulates human mesenchymal stem cell paracrine functions. *Stem Cells Translational Medicine*.

[43] Saller M. M., Prall W. C., Docheva D. (2012). Increased stemness and migration of human mesenchymal stem cells in hypoxia is associated with altered integrin expression. *Biochemical and Biophysical Research Communications*.

[44] Kim D. S., Ko Y. J., Lee M. W. (2016). Effect of low oxygen tension on the biological characteristics of human bone marrow mesenchymal stem cells. *Cell Stress and Chaperones*.

[45] Tamama K., Kawasaki H., Kerpedjieva S. S., Guan J., Ganju R. K., Sen C. K. (2011). Differential roles of hypoxia inducible factor subunits in multipotential stromal cells under hypoxic condition. *Journal of Cellular Biochemistry*.

[46] Chen Q., Shou P., Zheng C. (2016). Fate decision of mesenchymal stem cells: adipocytes or osteoblasts?. *Cell Death and Differentiation*.

[47] Gregory C. A., Prockop D. J., Spees J. L. (2005). Non-hematopoietic bone marrow stem cells: molecular control of expansion and differentiation. *Experimental Cell Research*.

[48] Holzwarth C., Vaegler M., Gieseke F. (2010). Low physiologic oxygen tensions reduce proliferation and differentiation of human multipotent mesenchymal stromal cells. *BMC Cell Biology*.

[49] Yun Z., Maecker H. L., Johnson R. S., Giaccia A. J. (2002). Inhibition of *PPARγ2* gene expression by the HIF-1-regulated gene *DEC1/Stra13*: a mechanism for regulation of adipogenesis by hypoxia. *Developmental Cell*.

[50] Fehrer C., Brunauer R., Laschober G. (2007). Reduced oxygen tension attenuates differentiation capacity of human mesenchymal stem cells and prolongs their lifespan. *Aging Cell*.

[51] Larsen S., Lewis I. D. (2011). Potential therapeutic applications of mesenchymal stromal cells. *Pathology*.

[52] Boregowda S. V., Krishnappa V., Phinney D. G. (2016). Isolation of mouse bone marrow mesenchymal stem cells. *Methods in Molecular Biology*.

[53] Nadri S., Soleimani M. (2007). Isolation murine mesenchymal stem cells by positive selection. *In Vitro Cellular & Developmental Biology - Animal*.

[54] Li H., Ghazanfari R., Zacharaki D., Lim H. C., Scheding S. (2016). Isolation and characterization of primary bone marrow mesenchymal stromal cells. *Annals of the New York Academy of Sciences*.

[55] Bara J. J., Richards R. G., Alini M., Stoddart M. J. (2014). Concise review: bone marrow-derived mesenchymal stem cells change phenotype following *in vitro* culture: implications for basic research and the clinic. *Stem Cells*.

[56] Souza M. C., Silva J. D., Pádua T. A. (2015). Mesenchymal stromal cell therapy attenuated lung and kidney injury but not brain damage in experimental cerebral malaria. *Stem Cell Research & Therapy*.

[57] Akiyama K., Chen C., Wang D. (2012). Mesenchymal-stem-cell-induced immunoregulation involves FAS-ligand-/FAS-mediated T cell apoptosis. *Cell Stem Cell*.

[58] Jiqing C., Yaqin L., Yingyin L. (2015). BMP4 inhibits myogenic differentiation of bone marrow–derived mesenchymal stromal cells in mdx mice. *Cytotherapy*.

